# GEPIA2: an enhanced web server for large-scale expression profiling and interactive analysis

**DOI:** 10.1093/nar/gkz430

**Published:** 2019-05-22

**Authors:** Zefang Tang, Boxi Kang, Chenwei Li, Tianxiang Chen, Zemin Zhang

**Affiliations:** 1 School of Life Sciences and BIOPIC, Peking University, Beijing 100871, China; 2 Peking-Tsinghua Center for Life Sciences, Academy for Advanced Interdisciplinary Studies, Peking University, Beijing 100871, China; 3 Beijing Advanced Innovation Center for Genomics, Peking University, Beijing 100871, China

## Abstract

Introduced in 2017, the GEPIA (Gene Expression Profiling Interactive Analysis) web server has been a valuable and highly cited resource for gene expression analysis based on tumor and normal samples from the TCGA and the GTEx databases. Here, we present GEPIA2, an updated and enhanced version to provide insights with higher resolution and more functionalities. Featuring 198 619 isoforms and 84 cancer subtypes, GEPIA2 has extended gene expression quantification from the gene level to the transcript level, and supports analysis of a specific cancer subtype, and comparison between subtypes. In addition, GEPIA2 has adopted new analysis techniques of gene signature quantification inspired by single-cell sequencing studies, and provides customized analysis where users can upload their own RNA-seq data and compare them with TCGA and GTEx samples. We also offer an API for batch process and easy retrieval of the analysis results. The updated web server is publicly accessible at http://gepia2.cancer-pku.cn/.

## INTRODUCTION

Valuable cancer-related RNA sequencing resources like TCGA and GTEx (1–3) have provided ample opportunities for data mining and deeper understanding of gene functions. As these data became available to researchers, numerous methods and procedures for analyzing large gene expression datasets also emerged, some of which were later accepted as common practices for mining such data. Survival analysis, for example, allows the identification of links between gene expression levels and prognostic outcomes, and hence has been widely used for evaluating the clinical relevance of a given gene. To facilitate the intuitive and interactive application of such methods to the TCGA and the GTEx datasets, we initially developed the GEPIA web server in 2016 (4). Armed with functionalities such as differential expression analysis, survival analysis, and similar gene identification, GEPIA provided experimental biologists and clinicians with a handy tool to explore TCGA and GTEx datasets. GEPIA has aided the investigation of various genes in multiple cancer types (5,6), and led to the identification of potential biomarkers (7) and therapeutic targets (8,9).

As we continuously accommodate the feedbacks and requests from our users, we have identified new angles to explore the TCGA and GTEx resources, and have thus developed a much-enhanced new version of GEPIA, named GEPIA2. For example, alternative splicing, resulting in different isoform usages, has gained traction in cancer target finding (10). In GEPIA2, we utilized the UCSC Xena (11) recomputed data of TCGA and GTEx for 198,619 coding and a series of other non-coding transcripts, and developed new computational functionalities to explore such events. Additionally, cancers are often comprised by heterogeneous subtypes with distinct prognosis. For instance, MSI and MSS colorectal cancers respond differently to chemotherapy and immunotherapy (12). To better investigate the underlying biological mechanisms of different cancer subtypes, GEPIA2 allows users to fine-tune their analyses to focus on each of the 84 cancer subtypes, and to compare across different subtypes. Furthermore, with single-cell sequencing becoming widely available, new paradigms of analysis have surfaced. In particular, gene signatures are often used to interrogate the composition of tumor microenvironment (13) and the functional status of infiltrating immune content (14). Such techniques are also integrated in to GEPIA2 as we provide signature-based functionalities with a curated list of signatures for efficient investigation. Finally, GEPIA2 provides features to process user-uploaded expression data and compare such data with the large collection of TCGA and GTEx samples.

## METHODS AND IMPLEMENTATION

**Figure 1.**
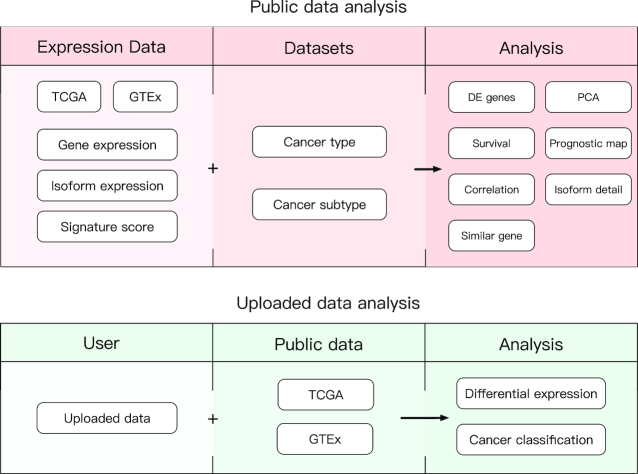
Schema describing the features of public data and uploaded data analyses in GEPIA2.

We added a collection of new features and upgraded multiple previous features in GEPIA2. To widen the analysis usage from gene level to isoform level and to better delineate different pathological status, isoform expression data and cancer subtype information are made available. The features in GEPIA2 are divided into two major topics: Expression Analysis and Custom Data Analysis. The Expression Analysis contains eight tabs: General, Differential Genes, Expression DIY, Survival Analysis, Isoform Details, Correlation Analysis, Similar Genes Detection and Dimensionality Reduction. Custom Data Analysis contains two tabs: Cancer Subtype Classifier and Expression Comparison (Figure 1). These features allow users to analyze the existing data and upload their own data for analysis based on different interactive functions. In addition to the web-based interface, we also provide a python package, to allow easy access of GEPIA2 analyses from a command-line environment. An overview for each new and upgraded feature is given in the following sections.

## NEW FEATURES

### Survival map

**Figure 2.**
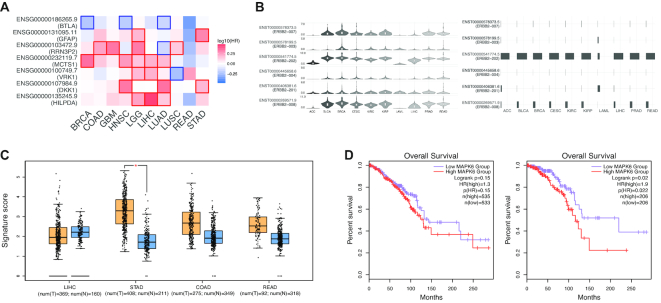
Examples of GEPIA2 outputs. (**A**) Users can check the prognostic impacts of gene or isoform expression level based on the survival heat map. The heat map shows the hazard ratios in logarithmic scale (log10) for different genes. The red and blue blocks denote higher and lower risks, respectively. The rectangles with frames mean the significant unfavorable and favorable results in prognostic analyses. (**B**) The violin-plots show the expression level (log_2_(TPM + 1)) of each isoform in a certain gene while the bar-plot panel present the isoform usage (from 0% to 100%) distribution. (**C**) GEPIA2 provides differential signature score analysis based on the box plots. The signature score is calculated by mean value of log_2_(TPM + 1) of each gene in Th1-like signature gene set. The orange box indicates the tumor samples while the blue one represents the normal tissues. The Th1-like signature score level in stomach tumors is significantly higher than normal tissues. (**D**) The overall survival analyses based on the cancer type and cancer subtype showed the significant prognostic impact of MAPK6 in Lumina A breast cancer subtype (left, *P* = 0.022) but not the whole breast cancer (right, *P* = 0.15).

GEPIA2 performs survival analyses based on gene or isoform expression levels. Given a list of custom cancer types, GEPIA2 would provide a heat map to show the survival analysis result based on multiple cancer types (Figure 2A). The red blocks denote higher and blue ones lower risk, with an increase in the gene or isoform expression. The blocks with darkened frames indicate statistical significance in prognostic analyses. Since gene survival analyses can be context-dependent (15), this function allows users to screen for the prognostic impact of a gene or an isoform across different cancer types.

### Isoform usage profiling

For a given gene and a list of custom cancer types, GEPIA2 generates the violin-plot and bar-plot panels for presenting the expression distribution and the isoform usage of each isoforms in the given gene (Figure 2B). Users can view the protein translation of each isoform in different cancer types and find the cancer specific isoform or isoform ‘switch’ event (16) in a certain cancer type. Meanwhile, GEPIA2 provides the isoform protein domain structure plots based on the prediction of Pfam for exhibiting the structural differences between each isoform.

### Uploaded expression data comparison

GEPIA2 bridges the gap between users’ custom data and the public cancer genomic data. This function enables users to upload their own data for performing differential analysis with the tumor or normal data from TCGA or GTEx projects. After the Quantile-normalization (17) or other two additional normalization strategies (inspired by GenomeSpace (18)) based on the uploaded dataset and the target dataset on the server, GEPIA2 performs the differential expression analysis and return result in boxplot.

### Cancer-subtype Classifier

With this function, users can accurately predict the cancer type or cancer-subtype of their uploaded samples based on the gene expression. The classifier is trained by TCGA expression data. For each sample, the probability of this sample belonging to each subtype would be listed below. We provide two modes to work with the classifier by choosing the actual cancer type first and predict for its corresponding subtypes or directly predict with the range of all cancer subtypes. The former way provides a more sensitive result, and the latter will be more useful for the identification of the cancer with unknown origin.

## UPGRADES

### Isoform analysis

Except for multiple gene comparison and dimensionality reduction functions, GEPIA2 allows users to perform all expression analyses at the isoform level (Table 1). Users can input an isoform symbol (e.g. ERBB2–001) or an Ensembl ID (e.g. ENST00000584601) to analyze an isoform of interest. Using different features, users can profile the isoform expression across different cancer types, obtain the differentially expressed isoforms, discover the prognostic isoforms and examine the correlation between two isoforms. Meanwhile, analyses based on both the gene and isoform levels could be performed to reveal the similarities or differences between them.

**Table 1.** Table describing the new features and improvements in the GEPIA2 visualization tools

		Gene	Isoform	Gene signature	Cancer subtype	Python package
General		✓	✓			
Differential genes		✓	✓			
Expression DIY	Gene expression profile	✓	✓			
	Box plots	✓	✓	✓	✓	✓
	Pathological stage plot	✓	✓			
	Multiple gene comparison	✓				
Survival analysis	Survival plots	✓	✓	✓	✓	✓
	Most differential survival genes	✓	✓			
	Survival Map	✓	✓			✓
Isoform details	Isoform usage		✓			
	Isoform structure		✓			
Correlation analysis		✓	✓	✓		✓
Similar genes detection		✓	✓	✓		✓
Dimensionality reduction		✓				

### Signature score

This function analyzes the prevalence of a gene signature in TCGA and GTEx samples. The mean value of the log_2_(TPM + 1) is used as the signature score. Based on the signature score, users can obtain the distribution of cell-type signature genes, such as effector T cell and exhausted T cell signature genes, across different cancer types. Meanwhile, GEPIA2 can calculate the correlation between two signatures and check their prognostic impact.

### Cancer subtypes

The subtype information on GEPIA2 allows users to select their subtype of interest for differential expression analysis or survival analysis. For the Box Plots feature, once users select the custom subtypes, they can select ‘Stacked’, ‘Separated’ or ‘Comparing Across Subtypes’ for different purposes. ‘Stacked’ indicates the approach to combine all the samples from selected subtypes prior to differential expression analysis with normal samples. ‘Separated’ means the method to perform differential expression analysis separately with normal samples. ‘Comparing Across Subtypes’ selection performs the differential expression analysis among the selected subtypes.

## DOCUMENTS AND AVAILABILITY

The GEPIA2 website is built in a similar architecture to GEPIA, using a combination of HTML5, JavaScript, and PHP for interactive controls. On the server side, GEPIA2 accesses and processes data using R and Perl scripts. We downloaded the TCGA and GTEx isoform expression data that are re-computed from raw RNA-Seq data by the UCSC Xena project (11) based on a uniform pipeline and collected different cancer subtypes information from TCGA papers. The datasets are stored in a MySQL relational database (version 5.7.17).

The GEPIA2 website is freely available to all users. GEPIA2 documentation and example pages are available and can be accessed by clicking the ‘Help’ link in the left navigation bar. Meanwhile, clicking the ‘Help’ button in each feature can open the collapsed tooltips, which contain the concise explanations and detail of each parameter. All plotting features in GEPIA2 are developed using similar packages and program as GEPIA (4). For more easily using, we use the dashboard to present the analysis in GEPIA2.

GEPIA2 python API provides users with access to GEPIA2’s analyses from a programming environment. Designed to perform multiple requests for our service, it boosts the efficiency of users by allowing batch process and streamlining results retrieval. The API is implemented in python language and we provide a tutorial for users to utilize the API. The python package provides access to 5 functionalities: boxplot, similar, correlation, survival and survival map.

Finally, we maintain a forum for users to discuss about the questions, problems or suggestions about GEPIA2.

## RESULTS AND DISCUSSIONS

GEPIA2 facilitates easy exploration of the large TCGA and GTEx datasets for experimental scientists. The availability of new data and new functionalities allow them to ask specific questions and test their hypotheses at a higher resolution. Although platforms like GenomeSpace allow users to perform similar analyses online, GEPIA2 provides a direct toolset to address these needs. Based on the new features and the improvements of the GEPIA2, users can focus on these important tasks:

### Immune-cell clusters analysis

Recently, single cell analyses based on the immune cells have gain tractions for different cancer types (14,19,20). Gene signatures of existing and novel immune-cell clusters have been identified. However, the existing data cannot present the expression distribution of a certain immune-cell cluster across cancer types. In this case, researchers can use GEPIA2 to easily obtain the immune-cell cluster signature profile in a pan-cancer manner. For example, one can readily find that Th1-like signature in stomach tumors is significantly higher than that in adjacent normal tissues (Figure 2C). Furthermore, researchers can validate the prognostic impact of these immune-cell clusters in a given cancer type.

### Subtype-specific prognostic analysis

*P*rognostic genes in different cancer types have been identified recently (21). These prognostic genes were identified based on a general cancer type rather than cancer subtype. However, different cancer subtypes in a cancer type can be distinct. For instance, triple-negative breast cancer has a ∼15% lower five-year survival rate compared to other breast cancer subtypes. It is thus desirable to perform prognostic analysis at a higher resolution. Based on the outputs of GEPIA2, we found that MAPK6 showed no association with breast cancer survival while it exhibited significant association with unfavorable prognostic outcome in Lumina A subtype (Figure 2D).

## CONCLUSIONS

We introduce GEPIA2 that significantly enhances the functionalities of GEPIA. By May 2019, GEPIA has been cited 414 times according to Google Scholar and has processed ∼400 000 analysis requests from ∼110 000 distinct IP addresses worldwide. In GEPIA2, the newly available isoform quantification and cancer subtype information will push the analyses to a higher resolution. New analysis methods like isoform usage profiling and custom data analysis arm users with a larger arsenal for deeper and wider interrogation of their genes of interest. With these new features, we believe GEPIA2 will be one of the most preferred tools for experimental biologists and clinicians to explore the big cancer genomics data.
